# Global Characteristics and Trends in Research on Ferroptosis: A Data-Driven Bibliometric Study

**DOI:** 10.1155/2022/8661864

**Published:** 2022-01-17

**Authors:** Xueting Dong, Yaochong Tan, Donglin Zhuang, Tingting Hu, Mingyi Zhao

**Affiliations:** ^1^Department of Pediatrics, The Third Xiangya Hospital, Central South University, Changsha, 410013 Hunan, China; ^2^Xiangya School of Medicine, Central South University, Changsha, 410013 Hunan, China; ^3^Guangdong Cardiovascular Institute, Guangdong Provincial People's Hospital, Guangdong Academy of Medical Sciences, Guangzhou, 510100 Guangdong, China; ^4^Department of Structural Heart Disease, National Center for Cardiovascular Disease, China & Fuwai Hospital, Chinese Academy of Medical Sciences & Peking Union Medical College, 100730 Beijing, China

## Abstract

Ferroptosis, an iron-dependent form of regulated cell death, has drawn an increasing amount of attention since it was first mentioned in 2012 and is found to play a significant role in the treatment of certain diseases. Our study is aimed at analysing the scientific output of ferroptosis research and at driving future research into novel publications. Publications focused on ferroptosis were retrieved from the SCI-EXPANDED database of the Web of Science Core Collection and were screened according to inclusion criteria. CiteSpace V and Microsoft Excel 2016 were used to evaluate and visualize the results, including generating network maps and analysing annual publications, country, category, references and cocited references, and keywords. As of October 1, 2021, a total of 1690 original articles related to ferroptosis were included, and the overall trend of the number of publications rapidly increased. Among the common categories in the field of ferroptosis, the most common category was biochemistry and molecular biology. Worldwide, China and the United States were the leading countries for research production. The retrieved 1690 publications received 44,650 citations, with an average of 26.42 citations per paper (October 1, 2021). By citation analysis, Scott J Dixon's article in 2012 was the most symbolic reference and the earliest publication in the field of ferroptosis, with the highest citation rate (2709 times). Among the most common keywords, most were related to the mechanisms and regulatory networks of ferroptosis. Furthermore, with accumulating evidence demonstrating the role of ferroptosis in cancers and other diseases, inducing ferroptosis in clinical treatment is becoming a new research focus that should be closely monitored.

## 1. Introduction

Ferroptosis is a newly identified form of nonapoptotic regulated cell death characterized by iron-dependent accumulation of lipid peroxides [1, 2]. Ferroptosis morphologically differs from other patterns of cell death and is mainly characterized by condensed mitochondrial membrane densities, a reduction in mitochondrial cristae, and outer mitochondrial membrane rupture [3–5]. Moreover, scholars have gradually revealed the process of ferroptosis, demonstrating the involvement of amino acids, lipids, and oxidation–reduction reactions [6–8]. The iron-dependent accumulation of lipid peroxides is regarded as a lethal event [9]. Furthermore, due to the characteristics affecting cell survival, ferroptosis plays an essential role in numerous diseases such as renal failure, tumours, nervous system diseases, and haematological system diseases [10–13]. In the past few years, topics on the difference between ferroptosis and other cell death types, the mechanism of ferroptosis, and the role of ferroptosis inducers and inhibitors have been widely studied [7, 14, 15]. We aimed to offer a systematic view of the evolution of ferroptosis with the intention of providing direction for further research.

Bibliometric sciences can not only offer a quantitative and statistical analysis of publications in specific areas but can also accurately uncover the most representative studies [16–18]. In addition, by presenting numerous data in the form of knowledge maps, researchers can comprehensively analyse the development of a discipline and intuitively understand frontier trends [19]. By means of CiteSpace, our investigations were based on the following aspects: annual publications, the network of categories and countries, references and cocited references analysis, and analysis of cooccurring keywords. The results could benefit researchers by shaping future research directions and providing references for policy formulation.

## 2. Material and Methods

### 2.1. Source Database and Data Collection

**Figure 1:**
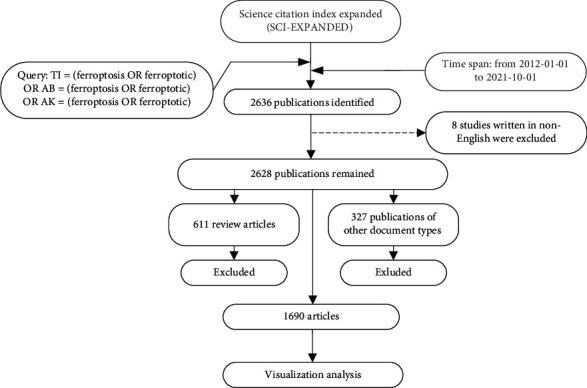
A study flow diagram of the retrieval strategy and selection process of the publications related to ferroptosis from SCI-EXPANDED.

In this study, we chose the Science Citation Index Expanded (SCI-EXPANDED) index of the Web of Science Core Collection (WoSCC) as the source database for data retrieval. The WoS Core Collection is a canonical online database providing a standardized and up-to-date dataset of reference for scientific research and analysis, among which SCI-EXPANDED is considered to be the most appropriate database for bibliometric analysis [20, 21]. Data were retrieved from the above database on October 1st, 2021. The following search strategy was employed: search query, TI = (ferroptosis OR ferroptotic) OR AK = (ferroptosis OR ferroptotic) OR AB = (ferroptosis OR ferroptotic). The timespan of the index date was set from January 1st, 2012, to October 1st, 2021. The language was restricted to English. For document types, only articles were included in the study, and all other document types were excluded. Nonetheless, to ensure the quality of retrieval, two independent authors were engaged in the search process, and both performed and confirmed the search query and results. Finally, a total of 1690 original articles were obtained. The dramatic increase in publications on ferroptosis in 2021 resulted in our including newly published articles in 2021 in the study. However, since we did not include the last two months of 2021 when setting the timespan, the 2021 data are not complete, and the analysis in this study does not represent the entire year. However, it does give insight into the latest research trends in the ferroptosis field, which will be further seen in the study context. The study flow diagram is shown in Figure 1.

### 2.2. Data Extraction and Analysis

The retrieved data were downloaded and exported into different formats for further analysis. Two authors performed the data extraction process independently. Information including titles, authors, institutions, abstracts, countries, source journals, total number of citations, publication year, keywords, and g-index was identified and extracted. The g-index is introduced as an improvement in the h-index, both of which are measures of productivity and the impact of a researcher [22]. If a set of data are ranked in decreasing order of the number of citations that they received, the g-index is the (unique) largest number such that the top *g* articles received (together) at least *g*^2^ citations [23, 24].

Visualization plays a significant role in bibliometric analysis because the collaborative networks, research hotspots, and trends of a new field can be intuitively observed through visualization maps. In this study, data analysis was performed using Microsoft Office Excel 2016 and CiteSpace v5.7.R2. Microsoft Office Excel 2016 was applied to manage the database and analyse the annual publication output and trend of ferroptosis publications. CiteSpace is a free software for visualizing data and analysing trends and patterns of the identified scientific literature as well as exploring the significant achievements, key research subjects, and outstanding researchers, institutions, and countries [25].

The retrieved data were put into the CiteSpace software to produce visualized maps to analyse the annual output counts, countries, top-cited references, keywords, and burst detection. Moreover, cocitations and coreferences were visualized to study cooperative relationships between identified publications. The parameters of CiteSpace were set as follows: time slicing (from 2012-01-01 to 2021-10-01), years per slice (1), links (strength = cosine; scope = within slices); selection criteria (g-index, *k* = 25), pruning (pathfinder + pruning sliced network + pruning the merged network); and the term source and node types are flexible parameters according to specific needs. Analyses of the methods included cooccurrence, clustering, and burst. The visualization diagram types included histograms, cluster views, timelines, and collinearity. All the clusters were labelled by keywords, and the log-likelihood rate (LLR) was used as the clustering algorithm. Synonyms were combined in the obtained analysis map according to the actual situation.

This study does not require ethical approval because it is a bibliometric analysis of existing publications, and no human subjects were enrolled.

## 3. Results

### 3.1. Annual Publication

**Figure 2:**
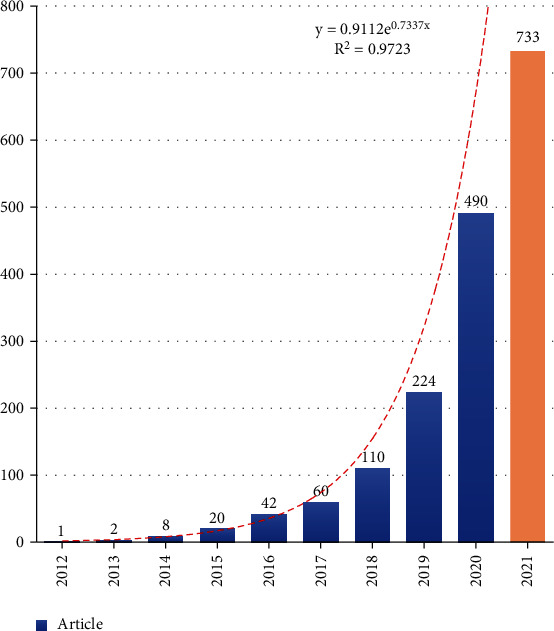
Annual frequency of publications in the field of ferroptosis. The data for 2021 is not complete.

A total of 1690 related publications were collected by searching for topic words.  Figure 2 illustrates the frequency distribution of the selected articles in line with the publication year. The number of applicable publications per year has been increasing annually from 2012 to 2021. The evolution of published papers on ferroptosis can be divided into two stages. The first stage was from 2012 to 2014, with a slow and steady rate of increase. The second stage was from 2015 to 2017, in which publication outputs increased gradually. The final stage was from 2018, during which there was a significant increase in publication counts from 2018 onwards. According to the calculation, the frequency of publications quadrupled in 2020 compared to 2018. Although the data for 2021 are not complete, it is estimated that the final number of publications in 2021 will increase rapidly.

### 3.2. Research Categories

**Figure 3:**
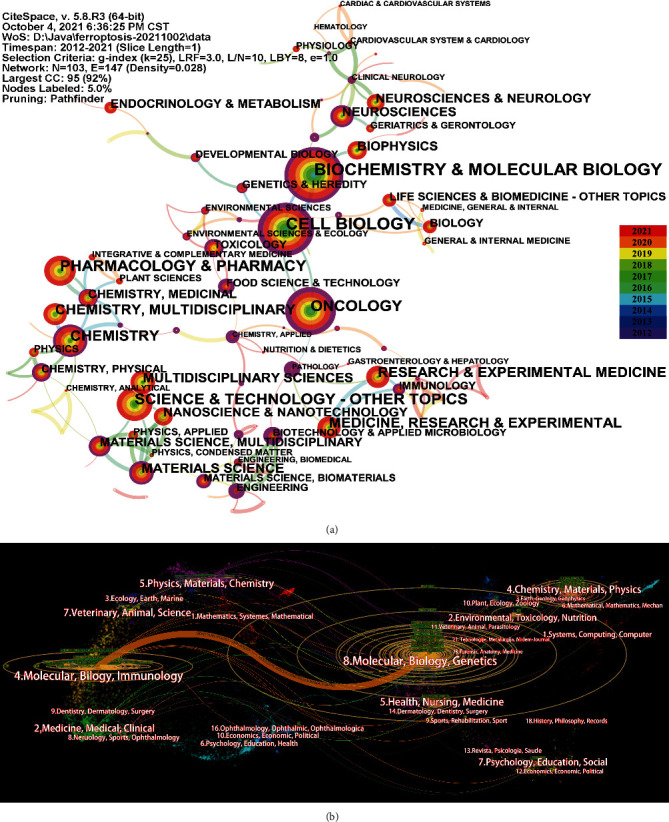
Category network map in the field of ferroptosis: (a) publication distribution among different categories; (b) dual-map overlay of journal publishing research.

Generating a category map using CiteSpace resulted in 103 nodes, meaning that a total 103 categories were involved in this research field (Figure 3(a)). The most frequently occurring category was biochemistry and molecular biology, which was the largest circle with a frequency of 400, followed by cell biology (315 publications). Other prominent categories included oncology (231 publications), pharmacology and pharmacy (195 publications), and science and technology (193 publications). Among those categories, pathology, cell biology, and oncology presented high centrality values of 0.83, 0.76, and 0.72, respectively. The top 10 categories related to ferroptosis in terms of count and centrality are shown in Supplementary Table 1. Furthermore, a dual-map overlay of the journals was designed to analyse the connection of subject categories to ferroptosis, as illustrated in Figure 3(b). The citation relationships are shown as spline waves, which are primarily rendered orange, green, blue, and purple. The spline curves start from the citing journals on the left and point to the cited journals on the right. These interactions present the flow and connections of different areas [26]. Only one primary citation path, coloured orange, was identified, indicating that the studies published in molecular, biology, and genetics journals were mainly cited by the studies published in molecular, biology, and immunology journals.

### 3.3. Contributions of Countries/Regions

**Figure 4:**
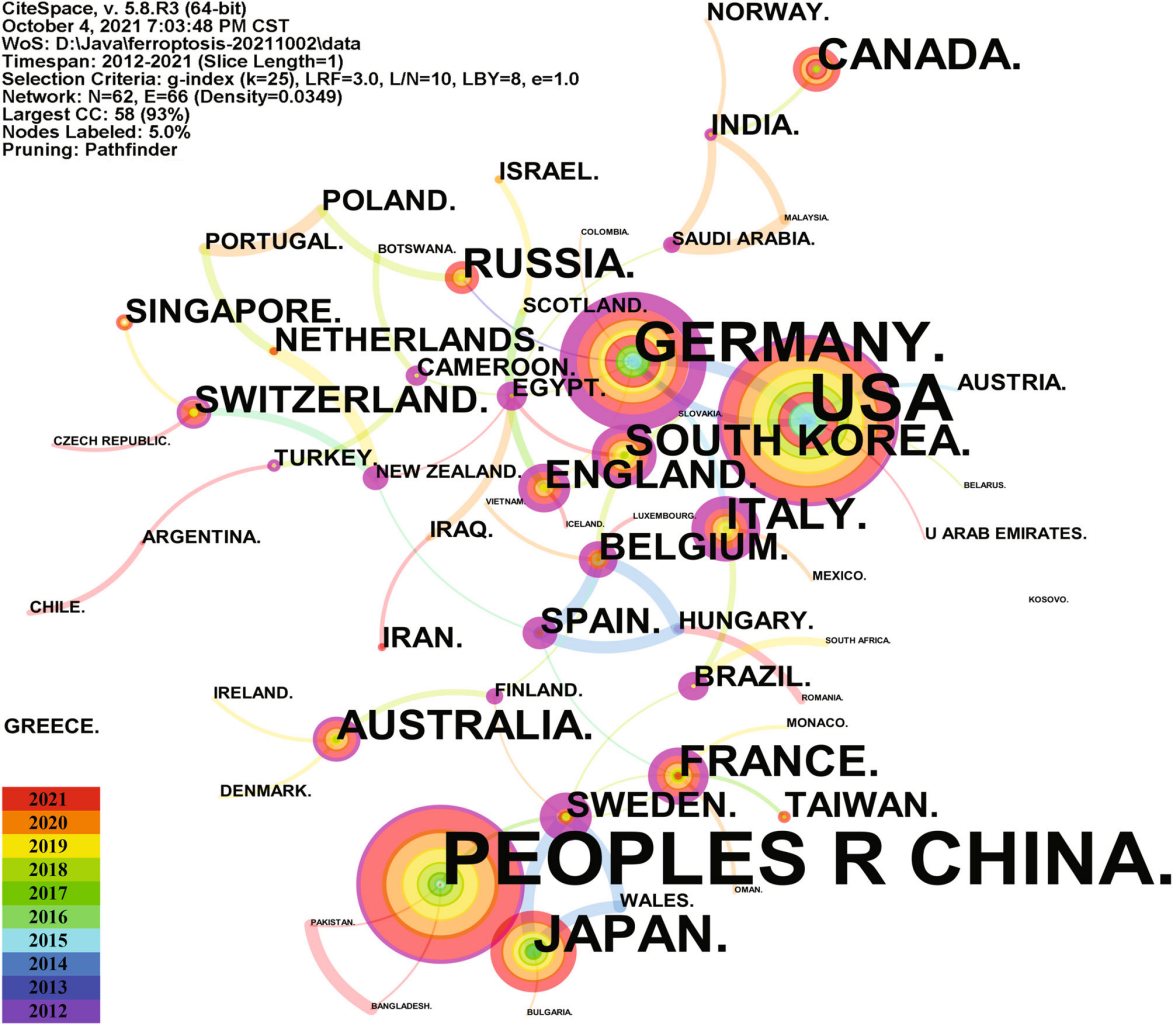
Distribution of countries/regions in the field of ferroptosis. Partial regions are independent of their countries when publishing articles, such as Taiwan.

The 1690 papers were published by research groups in 62 countries.  Figure 4 shows the network map and distribution of different countries or regions in which ferroptotic research was conducted. In Supplementary Table 2, the top 10 countries consisted of four European countries, three Asian countries, two North American countries, and one Oceanian country. China was the foremost productive country, with 975 publications, followed by the United States (428 publications, 25.33%), Germany (141 publications, 8.34%), Japan (101 publications, 5.98%), and South Korea (46 publications, 2.72%). Among the productive countries, the United States (2012), Germany (2013), France (2013), and Russia (2013) were the earliest countries to take up ferroptosis research. Germany, Sweden, and Egypt are coloured purple in Figure 4 based on their high betweenness centrality, which is usually regarded as a significant turning point that may lead to transformative discoveries and act as a bridge. The top 5 countries in terms of centrality were Germany (centrality = 0.73), Sweden (centrality = 0.66), Egypt (centrality = 0.55), New Zealand (centrality = 0.55), and Spain (centrality = 0.47).

### 3.4. References and Cocited References

#### 3.4.1. References

The retrieved 1690 documents received 44,650 citations in total, with an average of 26.42 citations per paper and an h-index of 100 (October 8th). The top 10 research articles related to ferroptosis citations are shown in decreasing order in Supplementary Table 3. There were a total of 8817 citations among the top 10 most cited research articles, accounting for 19.75% of the total citations for the 1690 retrieved documents in the framework of this study. The study by Dixon et al. titled “Ferroptosis: An Iron-Dependent Form of Nonapoptotic Cell Death,” published in 2012, is the most cited article, with a total number of citations of 2709 and the highest average number of citations of 270.9 per year, which was also where the term “ferroptosis” was coined. Following Dixon's study are “Regulation of Ferroptotic Cancer Cell Death by GPX4” by Yang WS in 2014, with a total citation of 1406 times and an average citation of 175.75 times per year, and Friedmann's study entitled “Inactivation of the ferroptosis regulator GPX4 triggers acute renal failure in mice,” with a total citation of 858 times and an average citation of 107.25 times per year. The top six original articles were published in *Cell* or *Nature* and its subjournals (*Nature* Cell Biology and *Nature* Chemical Biology).

#### 3.4.2. Cocited References

Cocitation analysis can help researchers find the common knowledge basis efficiently and conveniently among many studies [27, 28]. We input the retrieved 1690 articles to CiteSpace software and performed the cocitation analysis. After setting the parameters, a visualized cocitation map of the identified references was generated. Supplementary Table 4 lists the top 10 cocited references in terms of cocitation. The most cited reference was “Ferroptosis: A Regulated Cell Death Nexus Linking Metabolism, Redox Biology, and Disease,” published by Brent R Stockwell, 2017, with a cocitation count of 614. Following Brent R Stockwell were Y Xie, 2016, and Wan Seok Yang, 2016, which were cocited 386 and 316 times, respectively. All references in Supplementary Table 4 were cocited more than 150 times, and half of them were cocited over 250 times.

Furthermore, we explored the cocited references with strong citation bursts by using the CiteSpace software. References with citation bursts are defined as those cited frequently over a timespan. The top 10 cocited references with the strongest citation bursts are listed in ascending order according to the starting year of burst in Supplementary Table 5. References with cocitation bursts first emerged in 2013 due to Dixon's well-known publication in 2012. According to Supplementary Table 5, approximately 80% of the references had cocitation bursts between 2015 and 2018. The reference with the strongest citation strength (strength = 104.74) was “Regulation of ferroptotic cancer cell death by GPX4,” published in *Cell* by Wan Seok Yang et al. in 2014, followed by Scott J Dixon, 2012 (strength = 72.44), and Jose Pedro Friedmann Angeli, 2014 (strength = 65.71).

**Figure 5:**
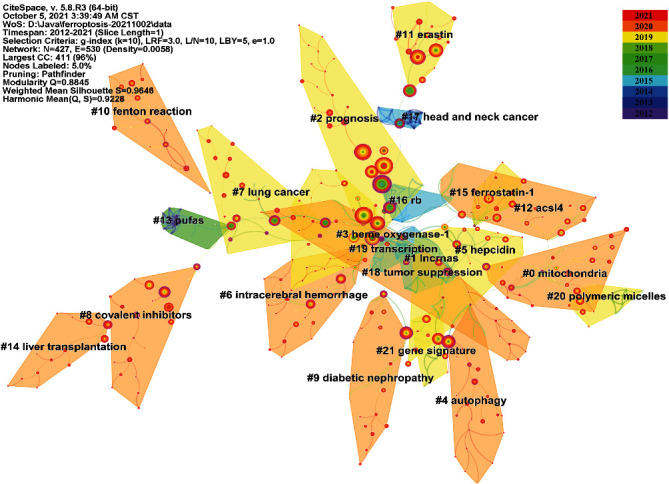
Cluster view of cocited references in ferroptosis research.

We also constructed a visualized map for clustering the cocited references with the aid of the CiteSpace clustering function. The retrieved documents were divided into 19 clusters through clustering analysis, as shown in Figure 5. The literature in each cluster is closely related to each other and coordinated in a specific field. The modularity *Q* score is 0.8884, while the mean silhouette value is 0.972, indicating that the clustering structure is stable and highly convincing. Notably, the Q/S value of the cocitation analysis is even higher than that of the coauthor analysis. The cluster nomenclature reflects the study frontiers in a certain field. In this study, the clusters were autogenerated and labelled by the log-likelihood ratio (LLR) algorithm. The largest cluster was #0, labelled “mitochondria,” followed by “IncRNA” (cluster #1), “prognosis” (cluster #2), and “heme oxygenase-1” (cluster #3). Other important clusters were “autophagy,” “hepcidin,” and “intracerebral haemorrhage,” which may represent a turn in the significance.

### 3.5. Key Topics of Research Hotpots

#### 3.5.1. Cooccurrence Analysis

**Figure 6:**
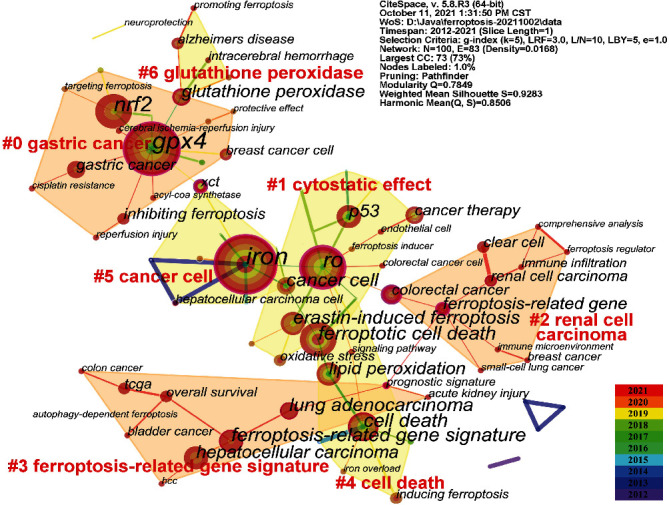
Keywords cooccurrence and cluster network in ferroptosis research.

Keywords are the core content of a document [29]. Using the CiteSpace software, a keyword cooccurrence map of ferroptosis was generated, as shown in Figure 6. Notably, the g-index was changed from *k* = 25 to *k* = 5 to obtain a better view of the distribution of keywords. As a result, a total of 100 keywords extracted from the title and author keywords of all 1690 publications were visualized and analysed with 110 links. As shown in Figure 6, the timespan was from 2012-01 to 2021-10, and the density value was 0.0168. The node represents the keyword, and the larger the circle is, the higher the frequency of the keyword. Each circle consists of a plurality of concentric colour rings, the colour of the ring represents the publication time of the literature containing the corresponding keywords, and the width of the ring indicates the number of documents published in the time unit. The thickness of the link between nodes indicates the frequency of the two keywords appearing in the literature, that is, the degree of association. After combining the synonyms and analogous keywords, the 10 most prominent keywords with the highest frequency were GPX4 (73 times), iron (73 times), RO (46 times), NRF2 (44 times), ferroptosis-related signature (27 times), cell death (26 times), ferroptotic cell death (25 times), erastin-induced ferroptosis (23 times), lung adenocarcinoma (22 times), and cancer cell (22 times). It could be inferred that the study of ferroptosis not only involves the manifestation and possible mechanisms, such as iron overload and the nrf2 pathway, but also refers to the exploration of indicators or regulators of ferroptosis, such as glutathione peroxidase 4 (GPX4) and erastin. In addition, ferroptosis-mediated cell death is widely involved in the occurrence and development of cancer, neurodegeneration, and other diseases.

#### 3.5.2. Cluster Analysis

Cluster analysis can demonstrate the knowledge structure of a certain field [30]. Clustering the included keywords and summarizing the themes of each cluster can help researchers understand the hotspots and ongoing trends of ferroptosis studies. According to the link strength of the cooccurrence terms, the network was classified into 7 clusters, which are shown in Figure 6. Terms are highly homogeneous in the same cluster. Cluster #0 is the largest cluster with 16 keywords: gastric cancer, cisplatin resistance, targeting ferroptosis, GPX4, neck cancer, RSL3, NRF2, breast cancer cell, protective effect, inhibiting ferroptosis, and so on. This cluster mainly focuses on gastric cancer. Cluster #1, which includes 14 keywords, is mainly related to the cytostatic effect: p53, endothelial cell, RO, erastin-induced ferroptosis, chemodynamic therapy, cancer therapy, cytostatic effect, epigenetic regulation, oxidative stress, ferroptosis inducer, and so on. The topic of cluster #2, which includes 10 keywords, is renal cell carcinoma, including immune microenvironment, small-cell lung cancer, breast cancer, clear cell, immune infiltration, comprehensive analysis, colorectal cancer, ferroptosis-related gene, ferroptosis regulator, and renal cell carcinoma. Cluster #3 is mainly related to cell death, with 10 keywords: ferroptosis-related gene signature, lung adenocarcinoma, hepatocellular carcinoma, TCGA, overall survival, bladder cancer, acute kidney injury, prognostic signature, colon cancer, and HCC. Cluster #4 focuses on cell death and includes 10 keywords: cell death, ferroptotic cell death, lipid peroxidation, inducing ferroptosis, signalling pathway, iron overload, mitochondrial dysfunction, acute myeloid leukaemia, diverse disease model, and acute lymphoblastic leukaemia cell. Cluster #5 is related to cancer cells and includes 9 keywords: iron, cancer cell, XCT, hepatocellular carcinoma cell, ferroptosis pathway, lipid peroxide, ferroptosis inducer erastin, artemisinin derivative, and iron-dependent cell death. The last cluster mainly deals with glutathione peroxidase and includes 5 keywords: glutathione peroxidase, Alzheimer's disease, intracerebral haemorrhage, promoting ferroptosis, and human cancer cells.

#### 3.5.3. Burst Detection

Keyword burst detection refers to detecting keywords with a high frequency of appearance in a certain period of time, which helps researchers analyse the evolution of ferroptosis research. In this paper, the time period was set from 2012-01 (since the concept of ferroptosis was coined in 2012) to the date when we performed the analysis. Through the burst test, the seven keywords identified with the strongest citation burst are shown in Supplementary Table 6 and include iron, cell death, cancer cells, glutathione peroxidase, ferroptotic cell death, neurodegeneration, and erastin-induced ferroptosis.

In general cancer cells had the strongest burst strength (strength = 5.18) followed by iron (strength = 3.58) and ferroptotic cell death (strength = 3.52). Dixon et al. introduced the concept of ferroptosis in 2012. Since then, ferroptosis has drawn increasing interest from scientific researchers. Iron dyshomeostasis is one the characteristics of ferroptosis. As an important inducing factor of ferroptosis, iron (strength = 3.58) was considered from 2015 to 2018. Cancer cells (strength = 5.18) were also consistently considered at almost the same time (from 2016 to 2019). The term ferroptotic cell death (strength = 3.52) had a citation burst during a short period from 2016 to 2017, while glutathione peroxidase (strength = 2.56) had a citation burst from 2016 to 2018. Cell death (strength = 2.56) became a burst word from 2017 until now. In recent years, oxidative stress (strength = 3.17) and ferroptosis induction (strength = 2.46) have become novel burst words, with citation burst years starting from 2019 to the present. These words will likely continue in the near future.

## 4. Discussion

### 4.1. General Information

The term “ferroptosis” was originally mentioned by Stockwell's group in 2012; thus, the literature was retrieved from WoS Core Collection from 2012 until now [2]. During the first two years after “ferroptosis” was officially coined, only three articles were published. The annual output grew steadily from 2014, which was related to the research of Yang's group in 2014 [8]. Their study was regarded as a significant and fundamental study and greatly advanced ferroptosis studies, with an extremely high citation frequency. Afterwards, with the fifth-highest citation frequency, the study of Doll's team in 2017 continued to boost the annual output [6]. Moreover, papers published from 2019 to the present day account for approximately 80 percent of all literature extracted, demonstrating that ferroptosis has been drawing increasing interest from scholars in recent years. Therefore, the topic of ferroptosis possibly remains a hotspot, and the number of publications is expected to increase in the coming years.

Among the common categories in the field of ferroptosis, the most important category was biochemistry and molecular biology. With the second-highest centrality and the second-highest frequency, the cross-domain of cell biology was exceedingly extensive, suggesting that it was a breakthrough in interdisciplinary research on ferroptosis. Moreover, with relatively high centrality, pathology, oncology, biotechnology, and applied microbiology were also breakthrough points. Since 2019, chemistry/applied food science and technology have gradually appeared in the scope of scholarly research and showed a high correlation with ferroptosis. For the dual-map overlay, Figure 3(b) illustrates that only one main citation path from molecular, biology, and genetics journals to molecular, biology, and immunology journals, suggesting that ferroptosis-related studies are focused on basic research.

Among the 62 countries and regions, only four of them have issued more than 100 papers, and it is worth noting that studies published by Chinese researchers accounted for more than half of the publications. However, the earliest research on ferroptosis was initially conducted by American scholars, Dixon and Stockwell's group, who conducted correlated research earlier than scholars from other regions of the world [2, 13, 31, 32]. Thus, the United States was one of the main driving forces on the academic impact reputation of ferroptosis research. It is undeniable that China was the only developing country among the top 5 productive countries. At present, the publication counts of China surpass those of the United States and rank first in the world in just a few years. Surprisingly, although the publication counts of Germany were lower than those of other countries, it had the closest cooperation with other countries and the highest centrality.

### 4.2. References and Cocited Reference Analysis

Highly cited references are classic studies that mainly address the mechanisms and potential pathways of ferroptosis. However, as research continues, new studies focusing on the regulation and manipulation of ferroptosis in human diseases are inevitably increasing in this field, for example, Kagan et al.'s publication and Sun et al.'s study [33, 34].

In Supplementary Table 4, the cocited reference with the highest citation is “Ferroptosis: A Regulated Cell Death Nexus Linking Metabolism, Redox Biology, and Disease,” published by Brent R Stockwell in 2017. In this paper, Brent R Stockwell completely summarized the biology of ferroptosis, links between ferroptosis and pathology, and potential applications in neoplastic diseases, which was one of the few significant reviews in the early study of ferroptosis. Moreover, the types of the top 3 cocited references with the most citations are “reviews.” Therefore, reviews with high quality are not only helpful for acquainting readers with ferroptosis but also provide novel research fields to scholars. Notably, six papers within the listed references in Supplementary Table 4 referred to the role of ferroptosis in tumour suppression. Considering that “cancer cell” serves as an essential keyword, we know these early studies laid the foundations for later research. The above papers are classic studies in the field of ferroptosis. In recent years, various studies have aroused new directions for researchers. These studies may not receive as many citations as earlier studies but still have great influence. For instance, in the paper, “Lymph protects metastasizing melanoma cells from ferroptosis” published in 2020, Ubellacker et al. observed higher levels of glutathione and oleic acid and less free iron in lymph, which reduces ferroptosis in metastases; thus, he answered the question of why cancer cells metastasize through the lymphatic system more easily than in blood [35]. As time passes by, these studies will be found and cited by an increasing number of researchers. The strongest cocitation bursts in Supplementary Table 5 show that the earliest burst began in 2013 due to Dixon's article published in 2012, with the second-highest burst strength. According to the results, approximately 80% of the references had cocitation bursts between 2015 and 2017. This may be related to the seven well-known articles published in 2014 in Supplementary Table 5.

Furthermore, the cocited reference cluster analysis in Figure 5 indicates that diseases (including hepcidin, prognosis, and intracerebral haemorrhage), cell biology (including mitochondria and autophagy), and mechanisms (including IncRNA and heme oxygenase-1) have been hotspots for ferroptosis-related scholars to date.

### 4.3. Keyword Analysis

Based on the existing knowledge, ferroptosis is a novel cell death pathway of nonapoptotic regulated necrosis [36]. The most significant characteristic of ferroptosis is lipid peroxidation, which is the consequence of multiple cellular metabolic dysfunctions, including exhaustion of glutathione, inactivation of GPX4, redox imbalance, iron overload, and other metabolic disorders, in addition to disturbance of cellular signalling pathways. The glutathione peroxidase family is a series of intracellular antioxidant enzymes, among which GPX4 has been confirmed to prevent lipid peroxidation and is thus closely related to the pathology of ferroptosis [37].

Accumulating evidence has demonstrated the contribution of ferroptosis to many organ injuries and neurodegenerative disorders. The most common type of organ injury is ischaemia/reperfusion injury of the kidney and heart [38–40]. The role of ferroptosis in neurodegenerative disorders, including Parkinson's disease, Alzheimer's disease, and stroke, during the ageing process has also been elucidated [41]. A majority of diseases affecting every human system involved ferroptosis to some degree. Notably, since researchers found the characteristic role of ferroptosis in inhibiting cancer growth and increasing the sensitivity of therapy-resistant cancer cells, the research network of ferroptosis in tumour suppression has increased. Hepatocellular carcinoma (HCC) is the most malignant and severe type of primary liver cancer worldwide and is the second leading cause of cancer-related death worldwide [42]. Although the underlying mechanisms of HCC are not fully understood, the discovery of ferroptosis brings new light to its diagnosis and treatment. Sorafenib, a first-line drug with multikinase inhibitory properties that is used as a reference treatment in advanced HCC, has been investigated intensively to induce ferroptosis in HCC cells [43, 44]. Mechanistically, the ferroptosis-inducing effect of sorafenib is due to upregulation of phosphorylation of ferroptosis-related genes or proteins, such as CAD protein (P27708), heavy chain ferritin (FTH1; P02794), and heme oxygenase 1 (HMOX1; P09601) [43]. However, sorafenib does not seem to be a bonafide ferroptosis inducer when applied to other cancers [45]. Overall, elucidating the role of ferroptosis in cancer pathogenesis and manipulating ferroptosis in the treatment of cancers has become a novel research interest in recent years. Furthermore, increasing evidence demonstrates the potential correlation between ferroptosis-related genes and human diseases, especially cancers. The ferroptosis-related gene signature shows great prognostic and diagnostic value in multiple diseases, such as hepatocellular carcinoma, gastric cancer, and melanoma [46–48]. These clues indicate that studies of ferroptosis and diseases have come down to the gene level. However, despite the clinical value, the exact mechanisms by which ferroptosis affects the tumour biology or pathogenesis of many other diseases are still enigmatic. Further investigation is expected to be conducted in the future.

As shown in the burst detection results, oxidative stress and ferroptosis induction are the latest burst terms in recent years, which suggest that these are the current research hotspots in this field. In recent years, studies of ferroptosis have extended to various aspects. In the first ten months in 2021, there have already been over 200 studies published in well-known journals. However, it did not become a burst term of ferroptosis in the study, similar to many other subfields in the ferroptosis study. Two possible reasons may contribute to the result: on the one hand, the research network has been broadened by researchers to a variety of research areas instead of several specific aspects; on the other hand, new publications still need time to be found and cited to form a new hotspot.

Combined with the results of keyword cooccurrence analysis and burst word detection, early studies mainly focused on the mechanism of ferroptosis, while in recent years, its clinical applications have become increasingly attractive. Thus, we can conclude that exploration of mechanisms has always been the mainstream of ferroptosis research, interpretation of ferroptosis in human diseases, and the application in tumour suppression are the hotspots and newly emerging research directions.

In recent years, the involvement of ferroptosis has been implicated in the occurrence and development of multiple human diseases; however, correlated studies are still very limited [49]. Ferroptosis-related gene signatures have been widely employed as prognostic or diagnostic tools in a variety of diseases in recent years, and inducing ferroptosis in cancer cells has been a novel direction for scientific researchers and health practitioners. With more attention paid to ferroptosis-related genes, RNAs, and proteins in the pathogenesis of cancers, it is appropriate to confer that manipulation of ferroptosis genes in cancer treatment might be of great therapeutic potential and a new hotspot in the future. It is believed that with the deepening of research on ferroptosis, more hotspots will appear in the near future, especially referring to the artificial regulation of ferroptosis in the treatment of human diseases.

## 5. Conclusion

In summary, ferroptosis research is in a rapid development stage. Annual publications on ferroptosis have been continually increasing since 2012, especially in the last three years. The most common category correlated with ferroptosis is biochemistry and molecular biology. China has made the most contribution to this field, while Germany has also played a significant role, with high centrality. Since ferroptosis is a newly discovered cell death modality, a majority of the keywords with the highest frequency in the field of ferroptosis are related to the mechanisms or pathways of ferroptosis. “Cancer cell” is the term with the strongest citation bursts, while “oxidative stress” and “inducing ferroptosis” are the latest two burst terms in recent years. With accumulating evidence demonstrating ferroptosis-related genes in the pathogenesis of multiple diseases, manipulation of ferroptosis-related genes (otherwise known as gene therapy) in clinical cancer treatment may soon become a new hotspot that should be closely monitored. Overall, compared to traditional reviews, we believe that the results of this study will provide objective insight into further research.

## Supplementary Materials

Supplementary 1 Supplementary Table 1: the top 10 categories related to ferroptosis in terms of count and centrality.

Supplementary 2 Supplementary Table 2: the top 10 countries/regions with the most publications for ferroptosis research.

Supplementary 3 Supplementary Table 3: the top 10 research articles of ferroptosis with the most citations.

Supplementary 4 Supplementary Table 4: the top 10 cocited references with the most citations in the field of ferroptosis.

Supplementary 5 Supplementary Table 5: the top 10 cocited references with the strongest cocitation bursts. The blue bars mean the reference had been published; the red bars mean cocitation burstiness.

Supplementary 6 Supplementary Table 6: the top 7 keywords with the strongest citation bursts.
