# Supplementary material for: Global Characteristics and Trends in Research on Ferroptosis: A Data-Driven Bibliometric Study
Source: Oxid Med Cell Longev. 2022 Jan 17;2022:8661864. doi: 10.1155/2022/8661864 (PMC8787456; doi:10.1155/2022/8661864)
Supplement: Supplementary 1 — Supplementary Table 1: the top 10 categories related to ferroptosis in terms of count and centrality. [file 8661864.f1.docx]

| **Rank** | **Category** | **Year** | **Count** | **Rank** | **Category** | **Year** | **Centrality** |
| --- | --- | --- | --- | --- | --- | --- | --- |
| 1 | BIOCHEMISTRY & MOLECULAR BIOLOGY | 2012 | 400 | 1 | PATHOLOGY | 2015 | 0.83 |
| 2 | CELL BIOLOGY | 2012 | 315 | 2 | CELL BIOLOGY | 2012 | 0.76 |
| 3 | ONCOLOGY | 2013 | 231 | 3 | ONCOLOGY | 2013 | 0.72 |
| 4 | PHARMACOLOGY & PHARMACY | 2015 | 195 | 4 | BIOTECHNOLOGY & APPLIED MICROBIOLOGY | 2017 | 0.7 |
| 5 | SCIENCE & TECHNOLOGY | 2014 | 193 | 5 | CHEMISTRY, APPLIED | 2019 | 0.67 |
| 6 | CHEMISTRY | 2014 | 159 | 6 | ENGINEERING | 2017 | 0.65 |
| 7 | RESEARCH & EXPERIMENTAL MEDICINE | 2015 | 132 | 7 | BIOCHEMISTRY & MOLECULAR BIOLOGY | 2012 | 0.57 |
| 8 | MEDICINE, RESEARCH & EXPERIMENTAL | 2015 | 132 | 8 | PSYCHIATRY | 2017 | 0.46 |
| 9 | CHEMISTRY, MULTIDISCIPLINARY | 2014 | 118 | 9 | ENGINEERING, CHEMICAL | 2018 | 0.42 |
| 10 | MULTIDISCIPLINARY SCIENCES | 2014 | 118 | 10 | FOOD SCIENCE & TECHNOLOGY | 2019 | 0.41 |

**Supplementary Table 1.** The top 10 categories related to ferroptosis in terms of count and centrality.
