# Supplementary material for: Global Characteristics and Trends in Research on Ferroptosis: A Data-Driven Bibliometric Study
Source: Oxid Med Cell Longev. 2022 Jan 17;2022:8661864. doi: 10.1155/2022/8661864 (PMC8787456; doi:10.1155/2022/8661864)
Supplement: Supplementary 2 — Supplementary Table 2: the top 10 countries/regions with the most publications for ferroptosis research. [file 8661864.f2.docx]

| **Rank** | **Continent** | **Country/Region** | **N (%)** | **Centrality** | **Year** |
| --- | --- | --- | --- | --- | --- |
| 1 | Asia | PEOPLES R CHINA | 975 (57.69%) | 0.12 | 2015 |
| 2 | North America | USA | 428 (25.33%) | 0.18 | 2012 |
| 3 | Europe | GERMANY | 141 (8.34%) | 0.73 | 2013 |
| 4 | Asia | JAPAN | 101 (5.98%) | 0.06 | 2014 |
| 5 | Asia | SOUTH KOREA | 46 (2.72%) | 0.29 | 2015 |
| 6 | Europe | FRANCE | 44 (2.60%) | 0.35 | 2013 |
| 7 | North America | CANADA | 43 (2.54%) | 0 | 2016 |
| 8 | Europe | ITALY | 41 (2.43%) | 0.45 | 2015 |
| 9 | Oceania | AUSTRALIA | 38 (2.25%) | 0.12 | 2017 |
| 10 | Europe | RUSSIA | 34 (2.01%) | 0.07 | 2013 |

**Supplementary Table 2.** The top 10 countries/regions with the most publications for ferroptosis research.
