# Supplementary material for: Global Characteristics and Trends in Research on Ferroptosis: A Data-Driven Bibliometric Study
Source: Oxid Med Cell Longev. 2022 Jan 17;2022:8661864. doi: 10.1155/2022/8661864 (PMC8787456; doi:10.1155/2022/8661864)
Supplement: Supplementary 3 — Supplementary Table 3: the top 10 research articles of ferroptosis with the most citations. [file 8661864.f3.docx]

| **Rank** | **Title** | **First author** | **Year** | **Total citations** | **Citation per year** | **Journal** |
| --- | --- | --- | --- | --- | --- | --- |
| 1 | Ferroptosis: An Iron-Dependent Form of Nonapoptotic Cell Death | Dixon SJ | 2012 | 2709 | 270.90 | CELL |
| 2 | Regulation of Ferroptotic Cancer Cell Death by GPX4 | Yang WS | 2014 | 1406 | 175.75 | CELL |
| 3 | Inactivation of the ferroptosis regulator Gpx4 triggers acute renal failure in mice | Friedmann Angeli JP | 2014 | 858 | 107.25 | NAT CELL BIOL |
| 4 | Ferroptosis as a p53-mediated activity during tumour suppression | Jiang L | 2015 | 734 | 104.86 | NATURE |
| 5 | ACSL4 dictates ferroptosis sensitivity by shaping cellular lipid composition | Doll S | 2017 | 617 | 123.40 | NAT CHEM BIOL |
| 6 | Oxidized arachidonic and adrenic PEs navigate cells to ferroptosis | Kagan VE | 2017 | 539 | 107.80 | NAT CHEM BIOL |
| 7 | Pharmacological inhibition of cystine-glutamate exchange induces endoplasmic reticulum stress and ferroptosis | Dixon SJ | 2014 | 522 | 65.25 | ELIFE |
| 8 | Glutaminolysis and Transferrin Regulate Ferroptosis | Gao M | 2015 | 507 | 72.43 | MOLECULAR CELL |
| 9 | Lipid peroxidation in cell death | Gaschler MM | 2017 | 468 | 93.60 | BIOCHEM BIOPHYS RES COM |
| 10 | Activation of the p62-Keap1-NRF2 pathway protects against ferroptosis in hepatocellular carcinoma cells | Sun X | 2016 | 457 | 76.17 | HEPATOLOGY |

**Supplementary Table 3.** The top 10 research articles of ferroptosis with the most citations.
