# Supplementary material for: Global Characteristics and Trends in Research on Ferroptosis: A Data-Driven Bibliometric Study
Source: Oxid Med Cell Longev. 2022 Jan 17;2022:8661864. doi: 10.1155/2022/8661864 (PMC8787456; doi:10.1155/2022/8661864)
Supplement: Supplementary 4 — Supplementary Table 4: the top 10 cocited references with the most citations in the field of ferroptosis. [file 8661864.f4.docx]

| **Rank** | **Co-citation** | **Title** | **Author** | **Year** | **Journal** | **Cluster ID** |
| --- | --- | --- | --- | --- | --- | --- |
| 1 | 614 | Ferroptosis: A Regulated Cell Death Nexus Linking Metabolism, Redox Biology, and Disease | Stockwell BR | 2017 | *Cell* | 2 |
| 2 | 386 | Ferroptosis: process and function | Xie Y | 2016 | *Cell Death And Differentiation* | 3 |
| 3 | 316 | Ferroptosis: Death by Lipid Peroxidation | Yang WS | 2016 | *Trends In Cell Biology* | 2 |
| 4 | 263 | ACSL4 dictates ferroptosis sensitivity by shaping cellular lipid composition | Doll S | 2017 | *Nature Chemical Biology* | 11 |
| 5 | 253 | Regulation of Ferroptotic Cancer Cell Death by GPX4 | Yang WS | 2014 | *Cell* | 2 |
| 6 | 188 | Activation of the p62-Keap1-NRF2 pathway protects against ferroptosis in hepatocellular carcinoma cells | Sun XF | 2016 | *Hepatology* | 3 |
| 7 | 186 | Oxidized arachidonic and adrenic PEs navigate cells to ferroptosis | Kagan VE | 2017 | *Nature Chemical Biology* | 11 |
| 8 | 185 | Mechanisms of ferroptosis | Cao JY | 2016 | *Cellular and Molecular Life Sciences* | 21 |
| 9 | 176 | Peroxidation of polyunsaturated fatty acids by lipoxygenases drives ferroptosis | Yang WS | 2016 | *Proceedings of the National Academy of Sciences* | 2 |
| 10 | 170 | Ferroptosis as a p53-mediated activity during tumour suppression | Jiang L | 2015 | *Nature* | 3 |

**Supplementary Table 4.** The top 10 cocited references with the most citations in the field of ferroptosis.
