# Supplementary material for: Global Characteristics and Trends in Research on Ferroptosis: A Data-Driven Bibliometric Study
Source: Oxid Med Cell Longev. 2022 Jan 17;2022:8661864. doi: 10.1155/2022/8661864 (PMC8787456; doi:10.1155/2022/8661864)
Supplement: Supplementary 5 — Supplementary Table 5: the top 10 cocited references with the strongest cocitation bursts. The blue bars mean the reference had been published; the red bars mean cocitation burstiness. [file 8661864.f5.docx]

| **Rank** | **References** | **Year** | **Strength** | **Begin** | **End** | **2012-2021** |
| --- | --- | --- | --- | --- | --- | --- |
| 1 | [Yang WS, 2014, CELL, V156, P317, DOI 10.1016/j.cell.2013.12.010, DOI](http://dx.doi.org/10.1016%2Fj.cell.2013.12.010) | 2014 | 104.74 | 2014 | 2019 | ▂▂▃▃▃▃▃▃▂▂ |
| 2 | [Dixon SJ, 2012, CELL, V149, P1060, DOI 10.1016/j.cell.2012.03.042, DOI](http://dx.doi.org/10.1016%2Fj.cell.2012.03.042) | 2012 | 72.44 | 2013 | 2017 | ▂▃▃▃▃▃▂▂▂▂ |
| 3 | [Angeli JPF, 2014, NAT CELL BIOL, V16, P1180, DOI 10.1038/ncb3064, DOI](http://dx.doi.org/10.1038%2Fncb3064) | 2014 | 65.71 | 2015 | 2019 | ▂▂▂▃▃▃▃▃▂▂ |
| 4 | [Dixon SJ, 2014, ELIFE, V3, P0, DOI 10.7554/eLife.02523, DOI](http://dx.doi.org/10.7554%2FeLife.02523) | 2014 | 41.07 | 2015 | 2019 | ▂▂▂▃▃▃▃▃▂▂ |
| 5 | [Skouta R, 2014, J AM CHEM SOC, V136, P4551, DOI 10.1021/ja411006a, DOI](http://dx.doi.org/10.1021%2Fja411006a) | 2014 | 38.68 | 2014 | 2019 | ▂▂▃▃▃▃▃▃▂▂ |
| 6 | [Linkermann A, 2014, P NATL ACAD SCI USA, V111, P16836, DOI 10.1073/pnas.1415518111, DOI](http://dx.doi.org/10.1073%2Fpnas.1415518111) | 2014 | 34.44 | 2015 | 2019 | ▂▂▂▃▃▃▃▃▂▂ |
| 7 | [Dixon SJ, 2014, NAT CHEM BIOL, V10, P9, DOI 10.1038/nchembio.1416, DOI](http://dx.doi.org/10.1038%2Fnchembio.1416) | 2014 | 32.52 | 2014 | 2019 | ▂▂▃▃▃▃▃▃▂▂ |
| 8 | [Jiang L, 2015, NATURE, V520, P57, DOI 10.1038/nature14344, DOI](http://dx.doi.org/10.1038%2Fnature14344) | 2015 | 22.58 | 2015 | 2018 | ▂▂▂▃▃▃▃▂▂▂ |
| 9 | [Gao MH, 2015, MOL CELL, V59, P298, DOI 10.1016/j.molcel.2015.06.011, DOI](http://dx.doi.org/10.1016%2Fj.molcel.2015.06.011) | 2015 | 21.3 | 2016 | 2021 | ▂▂▂▂▃▃▃▃▃▃ |
| 10 | [Vanden Berghe T, 2014, NAT REV MOL CELL BIO, V15, P134, DOI 10.1038/nrm3737, DOI](http://dx.doi.org/10.1038%2Fnrm3737) | 2014 | 12.68 | 2014 | 2018 | ▂▂▃▃▃▃▃▂▂▂ |

**Supplementary Table 5.** The top 10 cocited references with the strongest cocitation bursts. The blue bars mean the reference had been published; the red bars mean cocitation burstiness.
