# Supplementary material for: Global Characteristics and Trends in Research on Ferroptosis: A Data-Driven Bibliometric Study
Source: Oxid Med Cell Longev. 2022 Jan 17;2022:8661864. doi: 10.1155/2022/8661864 (PMC8787456; doi:10.1155/2022/8661864)
Supplement: Supplementary 6 — Supplementary Table 6: the top 7 keywords with the strongest citation bursts. [file 8661864.f6.docx]

| **Rank** | **Terms** | **Year** | **Strength** | **Begin** | **End** | **2012-2021** |
| --- | --- | --- | --- | --- | --- | --- |
| 1 | Iron | 2012 | 3.58 | 2015 | 2018 | ▂▂▂▃▃▃▃▂▂▂ |
| 2 | Cancer cell | 2012 | 5.18 | 2016 | 2019 | ▂▂▂▂▃▃▃▃▂▂ |
| 3 | Ferroptotic cell death | 2012 | 3.52 | 2016 | 2017 | ▂▂▂▂▃▃▂▂▂▂ |
| 4 | Glutathione peroxidase | 2012 | 2.56 | 2016 | 2018 | ▂▂▂▂▃▃▃▂▂▂ |
| 5 | Cell death | 2012 | 2.56 | 2017 | 2021 | ▂▂▂▂▂▃▃▃▃▃ |
| 6 | Oxidative stress | 2012 | 3.17 | 2019 | 2021 | ▂▂▂▂▂▂▂▃▃▃ |
| 7 | Inducing ferroptosis | 2012 | 4.26 | 2019 | 2021 | ▂▂▂▂▂▂▂▃▃▃ |

**Supplementary table 6.** The top 7 keywords with the strongest citation bursts.
